# Distribution characteristics of soil active organic carbon at different elevations and its effects on microbial communities in southeast Tibet

**DOI:** 10.3389/fmicb.2024.1458750

**Published:** 2024-10-23

**Authors:** Fanglin Ran, Chenni Zhou, Jianke Wang

**Affiliations:** ^1^Research Institute of Tibet Plateau Ecology, Tibet Agriculture and Animal Husbandry University, Nyingchi, China; ^2^Key Laboratory of Forest Ecology in Tibet Plateau, Ministry of Education, Nyingchi, China; ^3^National Key Station of Field Scientific Observation and Experiment, Nyingchi, China; ^4^Key Laboratory of Alpine Vegetation Ecological Security in Tibet, Nyingchi, China; ^5^Institute of Highland Forest Science, Chinese Academy of Forestry, Kunming, China; ^6^Research Center of Agricultural Economy, School of Economics, Sichuan University of Science and Engineering, Yibin, China

**Keywords:** soil active organic carbon component, microbial diversity, microbial composition and structure, elevation gradient, Tibet

## Abstract

Global mountain ecosystems have garnered significant attention due to their rich biodiversity and crucial ecological functions; however, there is a dearth of research on the variations in soil active organic carbon across altitudinal gradients and their impacts on microbial communities. In this study, soil samples at an altitude of 3,800 m to 4,400 m were collected from Sejira Mountain in the southeast Tibet, and soil active organic carbon components, soil microbial community diversity, composition and structure distribution and their relationships were systematically analyzed. The results revealed a non-linear relationship between the elevation and the contents of soil organic carbon (SOC) and easily oxidized organic carbon (ROC), with an initial increase followed by a subsequent decrease, reaching their peak at an altitude of 4,200 m. The Shannon diversity of bacteria exhibited a significant decrease with increasing altitude, whereas no significant change was observed in the diversity of fungi. The bacterial community primarily comprised Acidobacteria, Proteobacteria, Chloroflexi, and Actinobacteriota. Among them, the relative abundance of Proteobacteria exhibited a negative correlation with increasing altitude, whereas Actinobacteriota demonstrated a positive correlation with elevation. The fungal communities primarily consisted of Basidiomycota, Ascomycota, and Mortierellomycota, with Ascomycota prevailing at lower altitudes and Basidiomycota dominating at higher altitudes. The diversity and composition of bacterial communities were primarily influenced by altitude, SOC, ROC, and POC (particulate organic carbon). Soil carbon-to-nitrogen ratio (C/N), dissolved organic carbon (DOC), and available phosphorus (AP) emerged as key factors influencing fungal community diversity, while POC played a pivotal role in shaping the composition and structure of the fungal community. In conclusion, we believe that soil active organic carbon components had a greater impact on the bacterial community in the primary forest ecosystem in southeast Tibet with the elevation gradient increasing, which provided a theoretical basis for further understanding of the relationship between the microbial community and soil carbon cycle in the plateau mountain ecosystem under the background of climate change.

## 1 Introduction

Soil organic carbon (SOC) plays a pivotal role in the global carbon (C) cycle (Davidson and Janssens, 2006; Xia et al., 2014). The global SOC stock is estimated to range from approximately 1,500–2,400 Pg, which is greater than the combined amount present in atmosphere and vegetation combined (Lal et al., 2018; Ussiri and Lal, 2009). SOC is comprised of both highly stable C pools and active organic C components (Ramesh et al., 2019). Of these, the active C components are crucial for maintaining soil fertility and regulating the C cycle (Chen et al., 2022; Zhu and Shao, 2018; Barrow, 1993). The active organic C is the most readily available and, as a consequence, the most active fraction of SOC in the C cycle. This is due to its high solubility, mobility, and chemical instability, and its ability to rapidly decompose and transform (Lal et al., 2018). The active organic C components include rapidly oxidized carbon (ROC), dissolved organic carbon (DOC), microbial biomass carbon (MBC), and particulate organic carbon (POC) (Zheng et al., 2024). These components play different yet crucial roles in soil C metabolism: ROC is readily oxidized and plays a direct role in the C cycle; DOC is instrumental in the transport and bioavailability of C and nutrient; MBC reflects the activity of the microbial community and its capacity to store C; POC is derived primarily from plant residues and, though they decompose more rapidly than mineral organic C, they still play an important role in long-term C storage. At different altitude gradients, the distribution and response of active organic carbon components are significantly different, and are affected by changes in temperature, humidity and soil physicochemical properties (Liu et al., 2022; He et al., 2023; Xiang et al., 2022). Changes in altitude have a significant impact on the chemical processes and bioavailability of active organic carbon components, thereby influencing the accumulation and release of carbon in soil (Ramesh et al., 2019). Microbial communities play a pivotal role in maintaining the stability of soil organic carbon and its constituents (Fuhrman, 2009). Through their involvement in decomposing organic matter and participating in the carbon cycle, they directly influence the accumulation, release, and long-term storage of carbon in soils (Stockmann et al., 2013). Numerous studies have demonstrated that the diversity and functional activity of microbial communities play a crucial role in determining the turnover rate and stability of soil organic carbon (Breulmann et al., 2012). For instance, microbial communities exert control over soil fertility and carbon storage through the breakdown of active organic carbon components, such as sugars and amino acids (Condron et al., 2010). Moreover, microorganisms exhibit high sensitivity to environmental changes, enabling rapid responses to external conditions like temperature and humidity (Lau and Lennon, 2012), which directly impact their metabolic activity and biogeochemical processes, consequently influencing soil carbon loss and fixation. Notably, alterations in altitude gradients induce significant effects on both the structure and function of microbial communities (Bragazza et al., 2015; Frindte et al., 2019), thereby perturbing the dynamic equilibrium of active organic carbon components within soils. The low temperature and oxygen levels characteristic of high-altitude environments may suppress microbial activity, thereby facilitating the accumulation of soil organic carbon (SOC) and its active components (Dong et al., 2020). Moreover, bacterial and fungal communities exhibit distinct adaptation and survival strategies, with bacteria generally displaying a greater reliance on easily degradable organic carbon compared to fungi (Huang et al., 2021). Therefore, it is crucial to investigate the distribution characteristics of soil active organic carbon components and their relationship with microbial communities across different altitudinal gradients in order to enhance our understanding of soil carbon cycling and ecosystem functioning.

The Qinghai-Tibet Plateau, known as the “third pole” of the world, has become a sensitive and ecologically fragile area in terms of global climate change due to its complex terrain, variable climate conditions, and rich biodiversity (Wang et al., 2022; Yongxiu et al., 2020). Approximately 49 Pg of SOC is stored within the top meter soil layer on the Tibetan Plateau, accounting for 3.6% of the global soil carbon pool (Zhu and Shao, 2018; Genxu et al., 2002). Located in the southeastern part of the Qinghai-Tibet Plateau, Sejira Mountain serves as the watershed between the Niyang River and the Palong Zangbo River. It represents a significant geographical location and plays a crucial role in biodiversity conservation and ecosystem services (Feng et al., 2023; Fu et al., 2023a,b). In recent years, with advancements in microbial molecular technology, an increasing number of studies have been conducted on soil microorganisms across different altitudes and ecological types within the Qinghai-Tibet Plateau (Fu et al., 2023a,b; Wang et al., 2020). However, investigating soil microorganisms in Mount Sejira encounters several challenges including limited regional spatial sampling scale, insufficient continuity of sampling sites, and high heterogeneity of soil composition and microclimate. These factors pose difficulties for existing studies to fully unravel the complexity and functionality of these microbial communities (Chen et al., 2022). Therefore, it is imperative to conduct a systematic investigation into the distribution characteristics of soil active organic carbon components and their impact on soil microbial communities along different altitude gradients in Sejira Mountain. The primary objective of this study is to elucidate the variations in soil microbial community along an altitude gradient and explore the direct or potential relationship between these changes and soil active organic carbon components. By examining the alterations in soil active organic carbon components with increasing altitude, we aim to further comprehend the role of these components in the carbon cycle, as well as providing evidence to support the mechanism through which soil active organic carbon and its constituents influence soil microbial communities within alpine ecosystems.

## 2 Materials and methods

### 2.1 Overview of the study area

The Sejira Mountain (E93°12′~95°35′, N29°10′~30°15′) is situated in southeastern Tibet, China, on the northwestern flank of the Great Bend of the Yarlung Zangbo River and represents a southern extension of the Nianqing Tanggula Mountain. Spanning an elevation range from 3,800 meters to 5,200 meters above sea level, this region experiences influences from the Indian Ocean monsoon system, resulting in a climate characterized by mild winters and moderate summers with distinct dry and wet seasons. The annual average temperature is −0.73°C, with the highest monthly average temperature recorded at 9.23°C and the lowest at −13.98°C (Wang et al., 2019). The average annual precipitation is 1134.1 mm, with an accompanying average annual evaporation of 544.0 mm, representing approximately 48.0% of the total precipitation recorded annually. The changes of soil temperature and humidity at different elevations are shown in Supplementary Figure S1. Soil The summer rainy season spans from June to September and contributes significantly, accounting for 75–82% of the yearly precipitation volume, while maintaining a consistent average annual evaporation rate of 544.0 mm. The predominant soil type in this region is acidic brown loam characterized by its considerable depth and pronounced humification process, resulting in a pH range between 4 and 6 (Xiao-lin et al., 2008). The topography of Sejira Mountain exhibits significant undulations, with a pronounced vertical spectrum and a diverse range of vegetation types. The forest coverage rate reaches 55%, encompassing various coniferous forests and deciduous broad-leaved forests. Notable examples include *Abies georgei* var. *smithii*, *Pinus densata*, *Picea likiangensis* var. *linzhiensis*, *Rhododendron* spp., as well as *Quercus semicarpifolia* and other hardleaved broad-leaved forests.

### 2.2 Collection and handling of soil samples

Within the study area, plots were systematically selected at intervals of 100 m above sea level within the altitude range of 3,800 m to 4,400 m (with the timberline located at an altitude of 4,400 m). Each plot consisted of three quadrates measuring 30 m × 30 m, and a minimum distance of 400 m was maintained between each quadrat. After removing the surface litter in the quadrat, soil samples from the top 10 cm were collected using a stainless steel soil drill with an inner diameter of 38 mm employing a five-point sampling method. The collected soil samples within each quadrat were thoroughly mixed, sealed, and stored. Subsequently, the fresh soil samples were transported to the laboratory under freezing conditions where they underwent sieving (2 mm) to remove visible rocks as well as any remnants of soil fauna and flora. DNA extraction was performed on selected frozen soil samples at −80°C while the remaining samples were air-dried and stored at room temperature.

### 2.3 Determination of soil physical and chemical properties

The soil available phosphorus (AP) and total phosphorus (TP) were quantified using the molybdenum-antimony resistance colorimetric method (Ba et al., 2020). The determination of soil nitrate nitrogen (NO_3_^−^-N) and ammonium nitrogen (NH_4_^+^-N) contents was performed using SmartChem 200 automatic analyzer (Kepu Man Analytical Instrument (Beijing) Co, Ltd., Beijing, China). Soil nitrite nitrogen (NO_2_^−^-N) was determined through copper-cadmium reduction-diazotization coupling colorimetry (Bao, 2000). The content of total potassium (TK) was determined by sodium hydroxide alkali fusion method, and the content of available potassium (AK) was determined by ammonium acetate extraction combined with flame spectrophotometry. Soil total nitrogen (TN) and soil organic carbon (SOC) contents were determined by Elementar Vario EL III, Germany (Song et al., 2015). Soil microbial biomass carbon (MBC), microbial biomass nitrogen (MBN) and microbial biomass phosphorus (MBP) were determined by chloroform fumigation extraction method (Wu et al., 2006). The content of readily oxidized organic carbon (ROC) was determined by potassium permanganate oxidation method (Barrow, 1993), and the content of particulate organic carbon (POC) was determined by referring to the method of Cambardella and Elliott (1992). The concentration of soil soluble organic carbon (DOC) was determined using the Curtin method (Curtin et al., 2006).

### 2.4 16S rRNA and ITS high-throughput sequencing

The total microbial DNA was extracted from 0.6 g of mixed soil samples using a rapid extraction kit. Subsequently, the concentration and purity of the extracted DNA were determined utilizing a micro-UV spectrophotometer, followed by storage at −80°C subsequent to successful detection. The total DNA of soil microorganisms was amplified by PCR and a sequencing library was established (Shu et al., 2018). PCR amplification was a 20 μL reaction system consisting of 4 μL 5 × FastPfu Buffer, 0.8 μL primer (5 μmol/L), 2 μL 2.5 mmol/L dNTPs, 2 μL template DNA, and 0.4 μL FastPfu polymerase. PCR reaction conditions included an initial predenaturation step at 95°C for 2 min, followed by denaturation at 95°C for 20 s, annealing at 55°C for 40 s, and extension at 72°C for 1 min, repeated for a total of 35 cycles. Finally, the reaction was further extended for an additional duration of 10 min at 72°C. Each sample underwent independent amplification three times, and the resulting PCR products were subsequently analyzed using agarose gel electrophoresis with a concentration of 2.0%. All PCR products from the same sample were then pooled together. The combined mixture was purified using the Beckman Coulter Agencourt AMPure XP Kit, and subsequently subjected to indexing for 16S rRNA and ITS high-throughput sequencing libraries. The V3V4–1 segment of bacterial 16S rRNA was sequenced using the forward primer sequence (5′-ACTCCTACGGGAGGCAGCA-3′) and reverse primer sequence (5′-GGACTACHVGGGTWTCTAAT-3′), respectively. Sequential analysis was performed on the forward primer sequence (5′-GCATCGATGAAGAACGCAGC-3′) and reverse primer sequence (5′-TCCTCCGCTTATTGATATGC-3′) of the ITS2-1 segment. Subsequently, the obtained sequencing data underwent inspection and processing for taxonomic analysis. High-throughput sequencing of 16S rRNA and ITS regions was conducted by Guangdong MeGG Gene Technology Co., LTD.

### 2.5 Data analysis

The data analysis and visualization in this study were conducted using R (4.3.2). The distribution characteristics of soil active organic carbon components at different elevations were analyzed through linear or nonlinear fitting methods. Other physical and chemical properties of the soil were examined using one-way analysis of variance (ANOVA), and multiple comparisons were performed using Duncan’s new complex range method (*p* < 0.05). The sequencing data were processed using Mothur (version v.1.30) software to assess the Alpha diversity index of the sample, which included the Chao1 index for estimating the number of operational taxonomic units (OTUs) present in the sample, as well as the Shannon and Coverage indices for evaluating microbial diversity. The metaMDS function from the “vegan” package in R was employed to examine the dissimilarities in bacterial and fungal community structure across various altitudes (Beta diversity), while the adonis2 function was utilized to quantify and assess the impacts of different altitudes on bacterial and fungal community structure. Additionally, a linear regression model was applied to investigate the relationship between active organic carbon components and the diversity of bacterial and fungal communities. The importance of soil active organic carbon components and other physicochemical properties of soil on bacterial and fungal community diversity was assessed by constructing a random Forest model using the “Random Forest” package’s random Forest function in R. Additionally, the effects of soil active organic carbon components and other physicochemical properties on bacterial and fungal community structure were analyzed using the cca function in R’s “vegan” package and the mantel_test function in the linkET package.

## 3 Results

### 3.1 Distribution characteristics of soil active organic carbon at different altitudes

**Figure 1:**
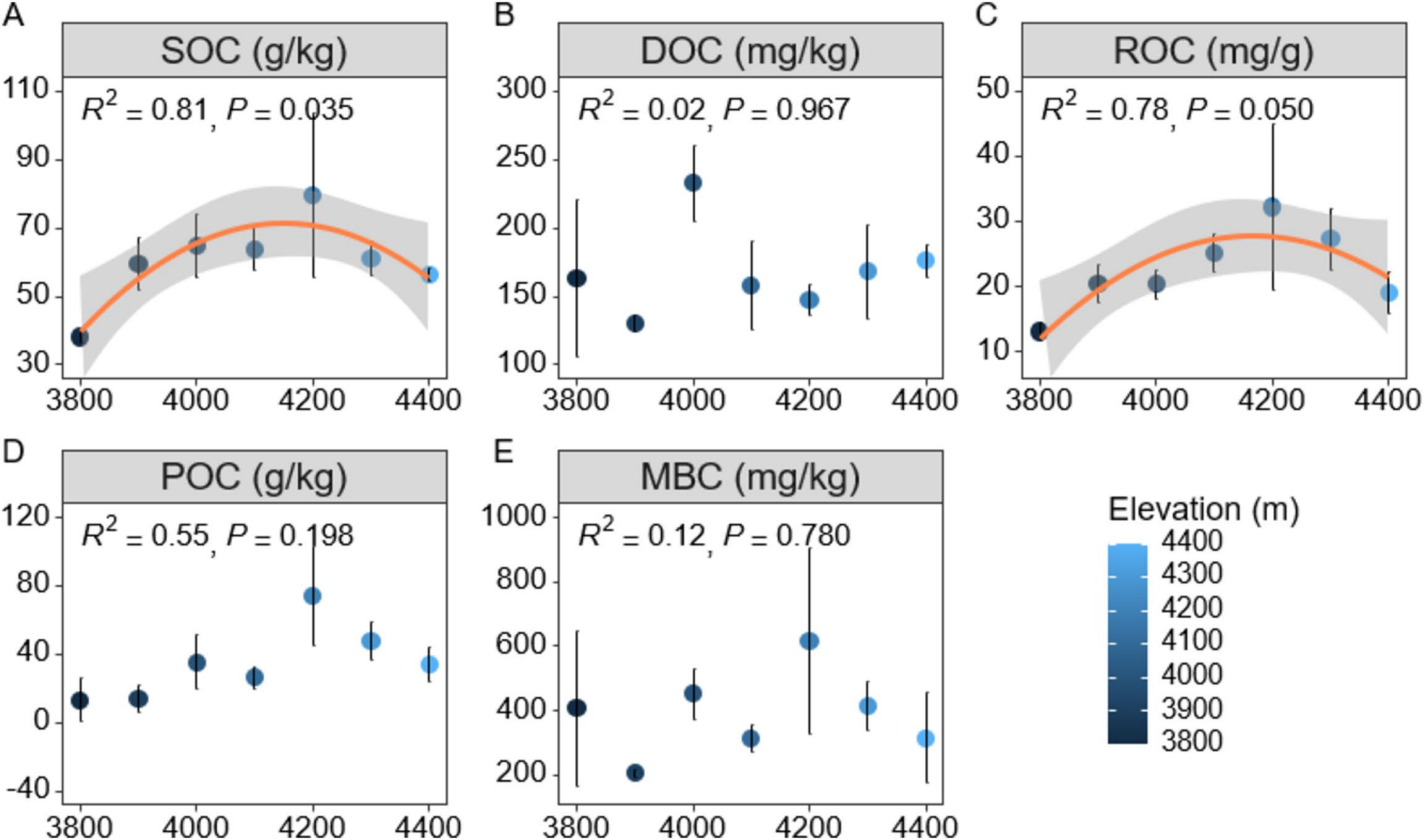
Distribution characteristics of soil active organic carbon components at different altitudes. **(A)** SOC (Soil Organic Carbon). **(B)** DOC (Dissolved Organic Carbon). **(C)** ROC (Readily Oxidized Organic Carbon). **(D)** POC (Particulate Organic Carbon). **(E)** MBC (Microbial Biomass Carbon). SOC represents the total organic carbon; DOC refers to dissolved organic compounds in soil water; ROC is the fraction of carbon that is easily oxidized; POC represents organic carbon attached to soil particles; MBC indicates the organic carbon in microbial biomass.

In order to gain a deeper understanding of soil carbon cycling in Mount Sejira, this study examined the vertical distribution characteristics of soil active organic carbon components at different elevations (Figure 1). The findings revealed that altitude had a significant impact on SOC (*p* < 0.01), with SOC content initially increasing and then decreasing as altitude increased, reaching its peak value of 79.64 g/kg at an elevation of 4,200 m (Figure 1A). Additionally, the responses of various soil active organic carbon components to changes in altitude exhibited notable differences (Figures 1B–D). Specifically, the response of ROC to altitude was found to be statistically significant (*p* < 0.05), exhibiting a decline after reaching its peak at an elevation of 4,200 m (Figures 1C,D). This decrease could potentially be attributed to reduced inputs of plant and animal residues at higher altitudes. Conversely, DOC, POC, and MBC components did not exhibit any significant changes with increasing elevation (*p* > 0.05) (Figures 1B,E). Correlation analysis further confirmed a strong positive association between SOC and other active organic carbon components (ROC, POC, and MBC) (*p* < 0.01) (Supplementary Figure S2). Notably, the correlation between SOC and ROC was the highest observed among these associations (*p* < 0.001), while the correlation between SOC and DOC was non-significant (*p* > 0.05) (Supplementary Figure S2). Additionally, no significant correlations were detected between DOC and any of the active organic carbon components including ROC, POC, or MBC.

The physical, chemical and biological properties of soil exhibited significant variations along different altitude gradients (Table 1). The NH+ 4-N content initially increased and then decreased with increasing altitude, reaching its lowest value of 2.38 mg/kg at an elevation of 4,300 m. At an elevation of 3,800 m, the AP content was found to be 1.8 times higher compared to that at 4,300 m. The TP content reached its minimum at both the forest line area and an elevation of 3,800 m, measuring 0.39 g/kg and 0.38 g/kg, respectively. In addition, the ratio of AK, NO- 3-N, TN in soil is the lowest at the low altitude, and increases with the increase of altitude, reaching the highest value at 4,200 m and then starting to decline. MBC/MBN and C/N in soils decreases with the increase of altitude. These results indicate that altitude significantly affects soil nutrient content, which usually increases first and then decreases with altitude, reflecting the influence of complex environmental gradients on soil nutrient dynamics.

**Table 1:** Effects of different elevation gradients on soil physical and chemical properties.

Altitude (m)	3,800	3,900	4,000	4,100	4,200	4,300	Timberline	*F*	*p*
Soil temperature (°C)	9.3 ± 0.4bc	11.7 ± 1.2ab	13.8 ± 1.0a	8.5 ± 0.7bc	9.8 ± 0.4bc	6.8 ± 0.5c	9.0 ± 0.5bc	5.5	0.02*
Soil moisture (%)	33.5 ± 0.4b	51.6 ± 1.0a	44.0 ± 1.7a	53.7 ± 1.4a	48.3 ± 3.7a	53.0 ± 4.0a	48.9 ± 1.2a	8.8	0.01*%
AP (mg/kg)	2.27 ± 0.41a	1.65 ± 0.35abc	2.15 ± 0.38ab	1.67 ± 0.37abc	1.65 ± 0.38abc	1.26 ± 0.23c	1.58 ± 0.24bc	3.06	0.04**
AK (g/kg)	0.06 ± 0.03ab	0.03 ± 0.01b	0.06 ± 0.01ab	0.08 ± 0.04a	0.09 ± 0.03a	0.07 ± 0.01ab	0.05 ± 0.03ab	1.67	0.20
NH+ 4-N (mg/kg) (mg/kg)	3.00 ± 0.19bcd	3.70 ± 1.88abcd	4.07 ± 0.58abc	4.61 ± 0.57ab	4.97 ± 0.52a	2.38 ± 0.55d	2.71 ± 0.56 cd	3.93	0.02*
NO- 3-N (mg/kg)	1.28 ± 0.84a	1.57 ± 0.81ab	2.14 ± 0.73ab	2.34 ± 0.07ab	2.59 ± 0.51a	1.78 ± 0.09ab	1.89 ± 0.19ab	1.95	0.14
NO- 2-N (mg/kg)	1.88 ± 1.00a	1.89 ± 0.20a	1.96 ± 0.20a	2.48 ± 0.48a	2.22 ± 0.31a	2.52 ± 0.51a	2.25 ± 0.61a	0.74	0.62
TP (g/kg)	0.64 ± 0.06a	0.39 ± 0.14c	0.57 ± 0.04a	0.55 ± 0.04ab	0.58 ± 0.05a	0.41 ± 0.09bc	0.38 ± 0.13c	4.32	0.01*
TK (g/kg)	7.88 ± 1.32a	6.33 ± 0.26b	7.63 ± 0.3ab	7.56 ± 0.97ab	7.54 ± 0.85ab	7.99 ± 0.45a	8.16 ± 0.67a	1.81	0.17
TN (g/kg)	2.19 ± 1.04bc	1.99 ± 0.55c	3.89 ± 0.57ab	3.20 ± 0.54abc	4.2 ± 1.51a	3.13 ± 1.16abc	2.52 ± 0.54abc	2.49	0.08
MBN (mg/kg)	53.33 ± 58.82a	20.69 ± 6.1a	49.81 ± 6.00a	28.329 ± 13.15a	49.7 ± 10.01a	31.81 ± 3.31a	40.4 ± 22.59a	0.76	0.61
MBP (mg/kg)	2.52 ± 2.05a	3.26 ± 2.30a	98.12 ± 153.04a	12.63 ± 2.31a	13.60 ± 10.79a	14.15 ± 3.17a	13.13 ± 6.01a	1.01	0.46
MBC/MBN	19.95 ± 10.67a	10.49 ± 3.06a	9.30 ± 2.85a	12.11 ± 3.40a	11.91 ± 3.89a	13.81 ± 3.48a	8.02 ± 0.74a	1.9	0.16
Soil C/N	22.52 ± 3.95b	32.02 ± 11.03a	16.66 ± 0.25b	20.12 ± 1.71b	19.27 ± 1.45b	21.16 ± 7.22b	20.56 ± 1.24b	2.54	0.07

### 3.2 Distribution characteristics of soil microbial community diversity and composition at different elevations

**Figure 2:**
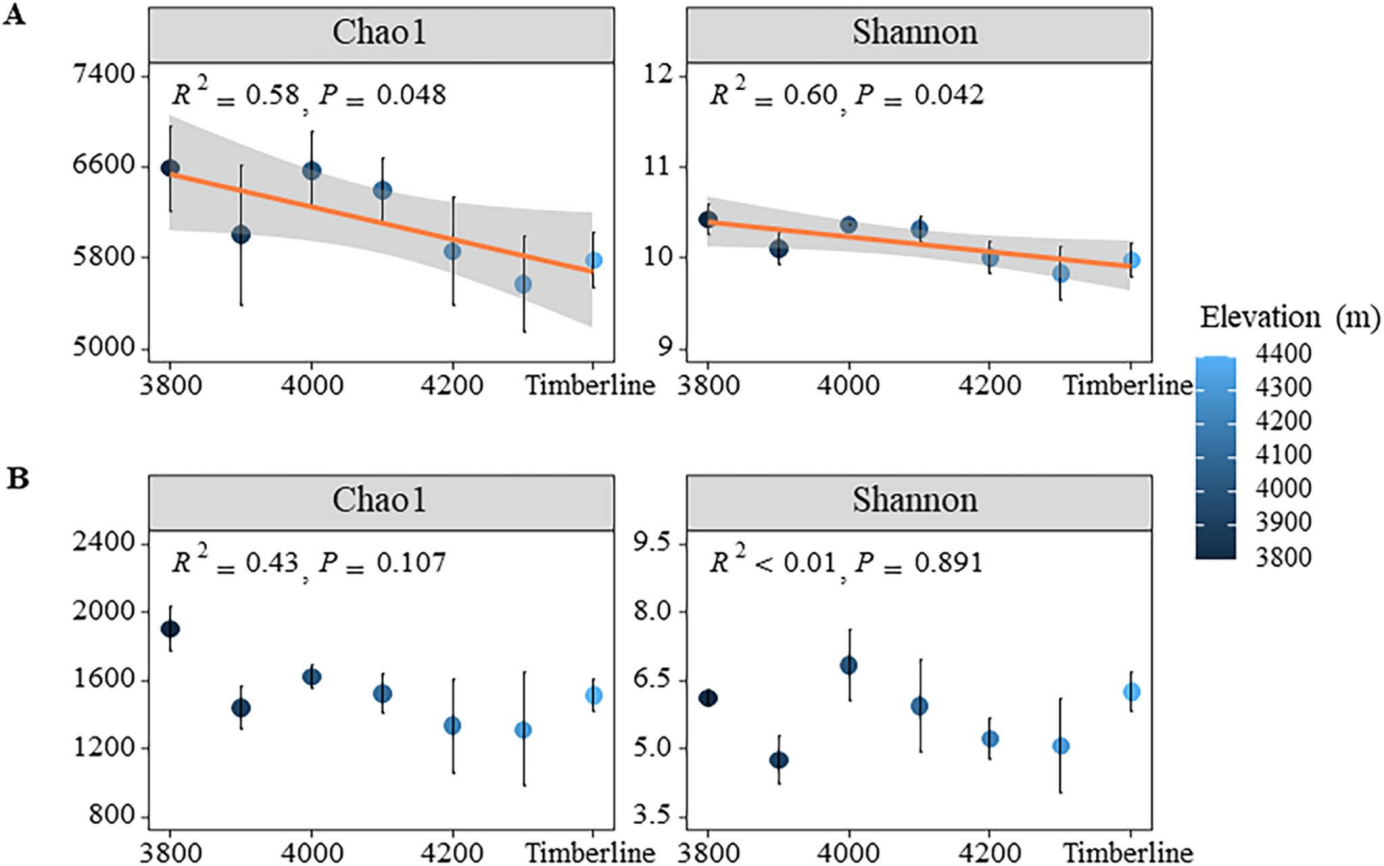
NMDS analysis of microbial community structure at different altitudes. **(A)** Bacteria **(B)** fungi.

Under varying altitude gradients, the Coverage index of bacteria and fungi in soil exceeded 0.98, indicating that the sequencing results accurately reflected the true composition of microorganisms in the samples (Supplementary Figure S2A). Overall, there was a significant decrease in both Shannon and Chao1 indices of bacterial communities with increasing elevation gradient (*p* < 0.05) (Figure 2A); specifically, the Shannon index decreased from 10.43 to 9.83 and the Chao1 index decreased from 6,591 to 5,574 (*p* < 0.01) (Supplementary Table S1). Additionally, the richness of bacterial communities also significantly decreased with elevation (*p* < 0.05), dropping from 5,648 at 3,800 m to 4,670 at 4,300 m, indicating a loss in bacterial diversity along the gradient. In contrast to bacteria, there were no significant changes observed in both Chao1 index and Shannon index of fungi with respect to elevation (*p* > 0.05) (Figure 2B). However, the richness of fungi did show significant variations (*p* < 0.05), with values decreasing from 1,422 at 3,800 m to 944 at 4,300 m, followed by a slight increase at the timberline (*p* = 0.02) (Supplementary Table S1).

**Figure 3:**
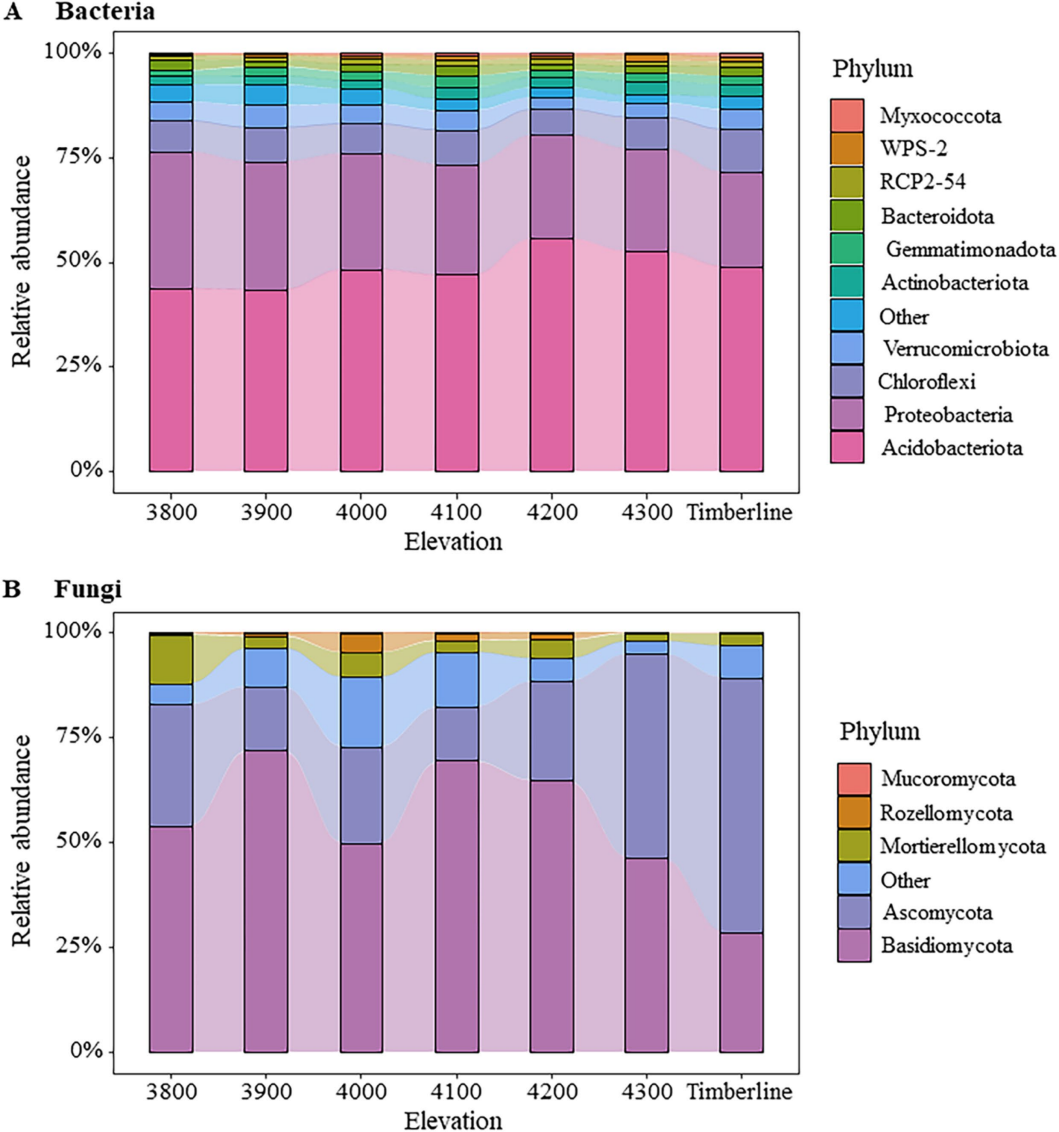
Changes in soil microbial community composition at different elevations. (A) Bacteria (B) fungi. The figure shows the relative abundance changes of the Top10 groups of bacteria and the Top5 groups of fungi. The remaining species groups of bacteria and fungi are combined into Other.

**Figure 4:**
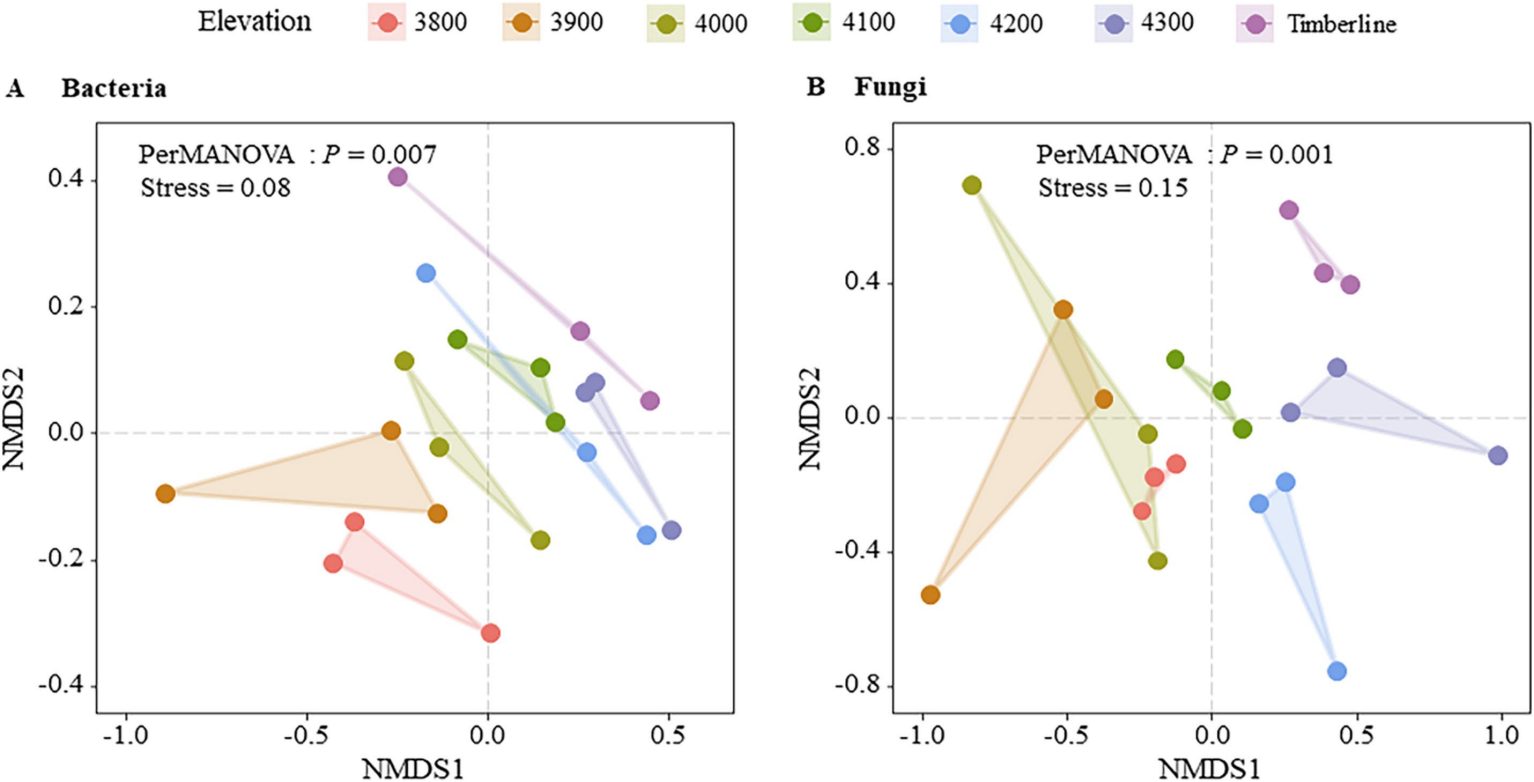
NMDS analysis of microbial community structure at different altitudes.

The microbial community composition exhibited significant variations along different altitude gradients (Figures 3, 4; Supplementary Table S2). At the bacterial phylum level, Acidobacteria (43.71 ~ 55.64%), Proteobacteria (22.42 ~ 32.52%), Chloroflexi (6.18 ~ 10.27%), Verrucomicrobiota (2.90 ~ 5.26%), and Actinobacteriota (1.90 ~ 3.35%) were identified as the dominant groups (Figure 3A). Notably, an elevation-dependent decline in the relative abundance of Proteobacteria was observed from 32.52 to 22.42% with increasing altitude, while a significant increase in the relative abundance of Actinobacteriota from 1.89 to 2.86% (*p* < 0.05) (Supplementary Table S2). At the phylum level, the dominant fungal groups were Basidiomycota (28.59 ~ 72.14%), Ascomycota (12.83 ~ 60.72%), and Mortierellomycota (1.86 ~ 11.70%) (Figure 3B). At an altitude of 3,900 m, Basidiomycota exhibited a significant dominance in the fungal community, accounting for 72.14% of the total relative abundance. The relative abundance of Basidiomycota gradually declined as the altitude increased to 4,200 m, while that of Ascomycota showed an increasing trend at this elevation range (*p* < 0.1) (Supplementary Table S2). Near the forest line, Ascomycota emerged as the prevailing group with a remarkable relative abundance reaching 60.72%. Furthermore, there was a substantial decrease in the relative abundance of Mortierellomycota (*p* < 0.01), from 11.70% at an altitude of 3,800 m to only 2.96% near the forest line (Supplementary Table S2).

Non-metric multidimensional scale analysis (NMDS) revealed significant effects of different altitude gradients on the structure of bacterial and fungal communities (*p* < 0.05) (Figure 4). The bacterial community structure showed significant changes with increasing altitude, as indicated by PERMANOVA (*p* = 0.007) (Supplementary Table S3). In particular, bacterial communities at higher altitudes (4,100 m–4,300 m) appeared to be more similar to each other, as shown by the clustering in the NMDS plot (Figure 4A). In contrast, the fungal community structure also exhibited significant changes with altitude (PERMANOVA, *p* = 0.001) (Supplementary Table S3), but no clear clustering pattern was observed across the altitudinal gradient (Figure 4B), indicating more dispersed distributions. The bacterial community structure exhibited significant variation with altitude (*p* = 0.019); however, the relationship between altitude and bacterial community similarity was weak (*R*^2^ = 0.01), as shown in the linear regression analysis (Figure 2B). In contrast, the fungal community structure did not show significant changes in similarity with altitude (*p* = 0.201), consistent with the more dispersed pattern observed in the NMDS results. These findings indicated that bacterial communities were more responsive to changes in altitude gradient than fungi in terms of diversity, composition, and structure.

### 3.3 Relationships between soil active organic carbon and microbial community diversity across different altitudes

**Figure 5:**
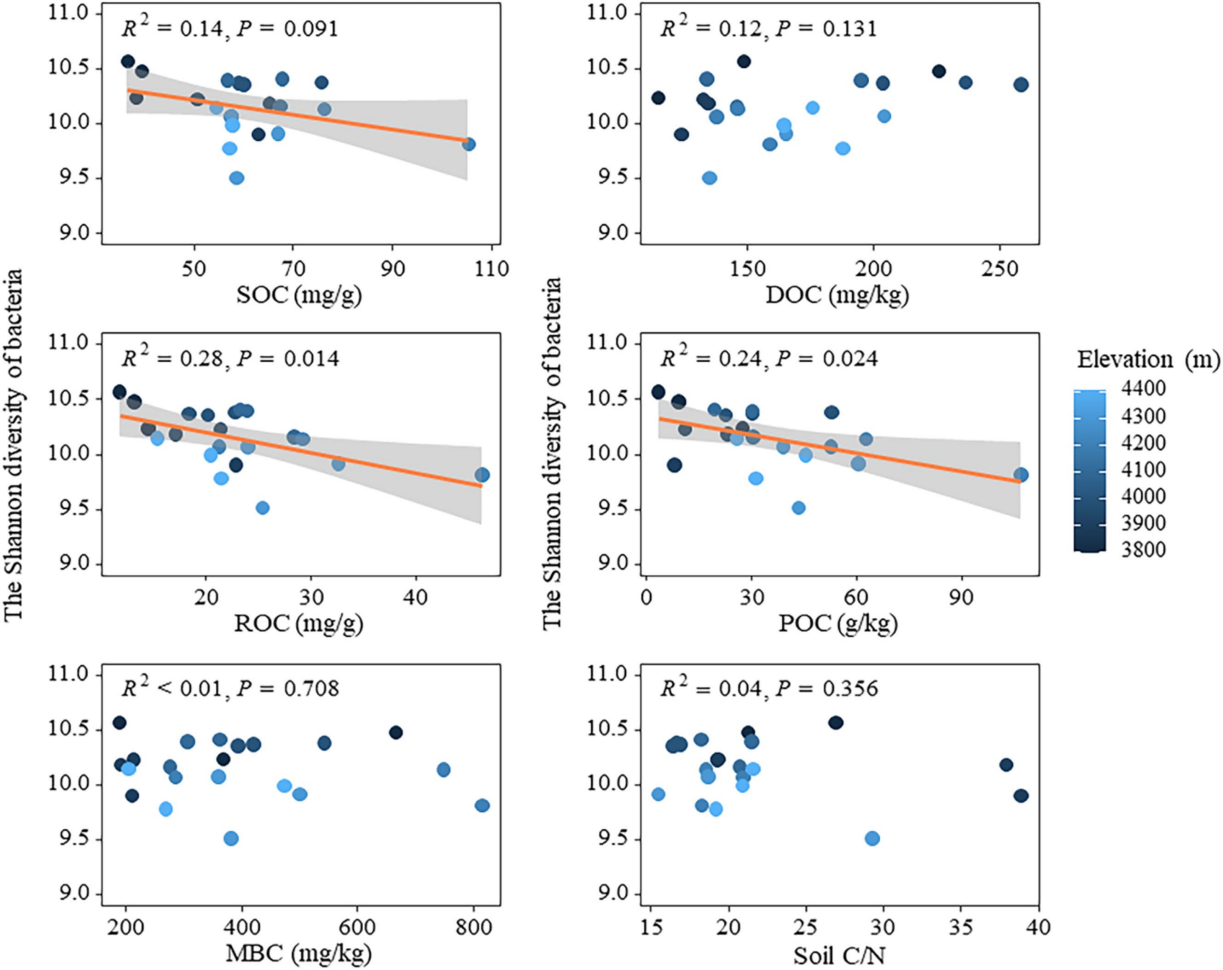
Relationships between soil active organic carbon components and bacterial diversity.

The relationship between bacterial and fungal community diversity and soil active organic carbon components was analyzed using linear regression models (Figure 5). The results revealed a weak negative impact of SOC on bacterial community diversity (*p* < 0.1), while both ROC and POC exhibited a significant negative influence on bacterial community diversity (*p* < 0.05) (Figure 5). The effects of DOC and MBC on bacterial community diversity did not show statistical significance (*p* > 0.05). Similarly, soil carbon to nitrogen ratio (C/N) had no significant impact on bacterial community diversity (Figure 5). Carbon fixation-related genes (cbbL and cbbM) were significantly and positively correlated with soil carbon pools (SOC, ROC, etc.) (Supplementary Figure S4). cbbL was highly correlated with SOC (*R* = 0.8, *p* < 0.01) and ROC (*R* = 0.72, *p* < 0.001), and cbbM was moderately correlated with SOC (*R* = 0.57, *p* < 0.01) and ROC (*R* = 0.45, *p* < 0.05) were moderately correlated. Genes involved in the degradation of complex organic matter, such as amylase and cellulase, also showed significant correlations with POC and ROC. Amylase was highly correlated with POC (*R* = 0.82, *p* < 0.05) and ROC (*R* = 0.75, *p* < 0.001), while cellulase showed the highest correlation with POC (*R* = 0.91, *p* < 0.001) and SOC (*R* = 0.83, *p* < 0.001) (Supplementary Figure S4). In addition, the random forest model was used to analyze the effects of active organic carbon components and other physicochemical properties on the diversity of bacterial and fungal communities (Supplementary Figure S5A). In terms of bacterial community diversity, altitude factors exhibited a higher relative importance, while environmental factors such as ROC and POC demonstrated a greater relative significance. Moreover, AP, DOC, and soil C/N ratio exerted a more pronounced influence on fungal community diversity. The findings not only suggested the distinct impacts of various active organic carbon components on the diversity of bacterial and fungal communities, but also demonstrated the pivotal role played by environmental factors such as elevation and soil physicochemical properties in governing these microbial communities through random forest models. Specifically, elevation and specific soil chemical properties including available phosphorus (AP), soluble organic carbon (DOC), and C/N exerted significant influences on maintaining or altering microbial community structure, highlighting the intricate dynamics of diversity in microecosystems and their susceptibility to environmental changes.

**Figure 6:**
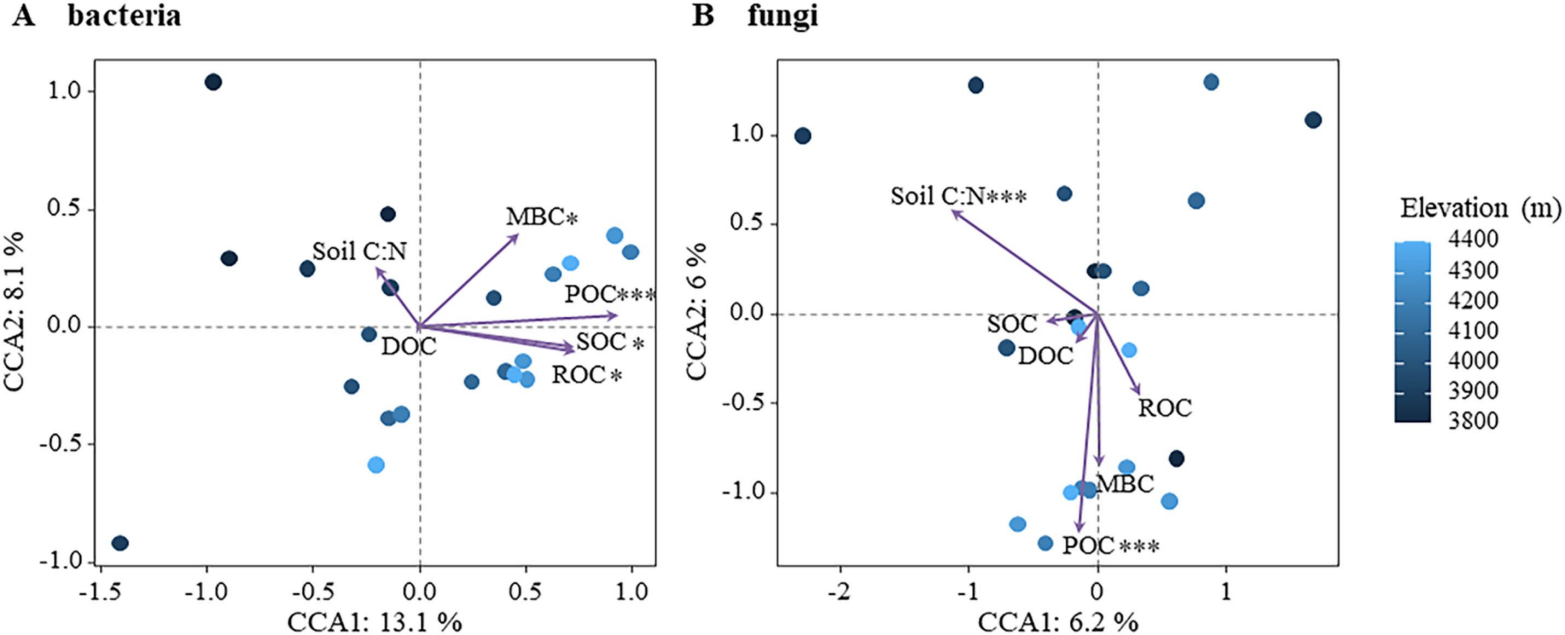
Effects of soil active organic carbon components on microbial community structure. (A) Bacteria (B) fungi.

The effects of active soil organic carbon components on bacterial and fungal community structure were further assessed using Canonical Correspondence Analysis (CCA), as shown in Figure 6. CCA analysis revealed that 21.2% of the variation in bacterial community structure could be explained, with CCA-1 accounting for 13.1% and CCA-2 accounting for 8.1%. All components including SOC, ROC, POC, and MBC exhibited significant impacts on bacterial community structure, with POC being identified as the most influential factor (Figure 6A; Supplementary Figure S5). In contrast, 12.2% of the variation in fungal community structure was explained, with the first main axis (CCA-1) and second main axis (CCA-2) explaining 6.2 and 6.0%, respectively. The fungal community structure was significantly affected by soil C/N and POC (Figure 6B; Supplementary Figure S5). In addition, the relationship between other physicochemical and bacterial and fungal communities in the soil was analyzed by Mantel test (Supplementary Figure S6). The bacterial community was significantly affected by altitude, SOC, POC and MBC, while the fungal community was significantly affected by altitude, NO_3_^−^-N and NO_2_^−^-N. These findings demonstrated that distinct active organic carbon components and soil C/N ratios exerted differential influences on the community structure of bacteria and fungi, highlighting the heightened sensitivity of bacteria toward soil active organic carbon components compared to fungi. The analysis revealed that soil moisture showed significant variation with elevation (*F* = 8.831, *p* < 0.01), with lower values at 3,800 m and relatively stable values between 4,000 m and the timberline. Similarly, soil temperature decreased significantly with elevation (*F* = 5.508, *p* < 0.05), especially beyond 4,000 m. However, neither soil temperature nor moisture varied with altitude in a significant pattern, nor were they significantly related to soil organic carbon and its reactive organic carbon fractions, nor to microbial communities (Supplementary Figure S6).

## 4 Discussion

### 4.1 Impacts of altitude gradient on soil active organic carbon and its components

The distribution of soil organic carbon (SOC) in the plateau is influenced by various environmental factors, particularly changes in elevation (Liu et al., 2011; Yang et al., 2008). In this study, we observed a non-linear relationship between altitude and SOC content, with an initial increase followed by a subsequent decrease, reaching its peak at an altitude of 4,200 m (Figure 1A). This pattern may be attributed to alterations in environmental variables such as soil temperature and moisture. At high altitudes, lower temperatures may inhibit microbial activity and slow down the decomposition rate of SOC, while increased humidity may improve the hydrolytic stability of SOC (Gu et al., 2019), which together promote the accumulation of SOC. However, as the altitude exceeded 4,200 m, SOC content began to decline (Figure 1A). The potential factors contributing to this phenomenon were as follows: Firstly, the elevation range reaches or exceeds the timberline, rendering the area unsuitable for tree growth and resulting in a substantial decline in aboveground vegetation biomass (Whittaker and Niering, 1975). As plant residues serve as the primary source of soil organic carbon, the reduction in vegetation biomass directly diminishes organic matter input and impacts SOC formation and accumulation (Paul, 2016). Secondly, diminished vegetation diversity and biomass weaken root activities while reducing root exudation (Semchenko et al., 2021), thereby decreasing the carbon supply to soil microbial communities and leading to a simplification of soil microbial community composition. This alteration reduced specific plant secretion-dependent microbial taxa, consequently affecting further transformation and decomposition of soil organic matter (Bhattacharyya et al., 2022).

ROC and POC exhibited a similar pattern of initial increase followed by decrease with elevation, reaching their maximum values at 4,200 m (Figures 1C,D). This trend is closely linked to the changes in environmental conditions associated with increasing altitude, particularly lower temperatures and higher humidity levels that significantly impact the biodegradation rate of ROC and POC (Tudi et al., 2021). The root structure and biomass allocation patterns of different vegetation types exerted significant influences on the active organic carbon components. For instance, herbaceous plants, which dominate at 4,200 m with nearly 40% coverage (Supplementary Figure S7), generate easily decomposable residues that rapidly convert into ROC (Kögel-Knabner, 2002; Yang et al., 2022). In contrast, shrub coverage increases consistently with elevation, reaching 80% at 4400 m (Supplementary Figure S7), contributing to POC accumulation due to their lignin-rich, slowly decomposing residues (Li et al., 2020). Easily decomposable residues may undergo swift conversion to ROC, while more recalcitrant residues tend to be progressively converted into POC. It is noteworthy that this study revealed no significant changes in DOC and MBC with increasing elevation (Figures 1B,E), suggesting a lower sensitivity of DOC and MBC components to altitude variations compared to ROC and POC, which contrasts with previous findings (Sheng et al., 2015; Zeng et al., 2022). The stability of DOC may be attributed to its refractory compounds in soil solution (Kalbitz et al., 2000; Kalbitz et al., 2003), while the resilience of MBC could be linked to the adaptability of microbial communities toward environmental fluctuations (Kalbitz et al., 2000; Kalbitz et al., 2003).

### 4.2 Impacts of altitude gradient and soil active organic carbon components on microbial community diversity

Previous studies have revealed different trends in soil microbial diversity with altitude, indicating that microbial distribution was affected by complex interactions between climate, topography, and biological factors (Fierer and Jackson, 2006; Siles and Margesin, 2016). The present study investigated soil microbial diversity and community structure across different altitudes above timberline of Sejira mountain. Our findings provided support for a portion of our hypothesis, indicating that soil bacterial and fungal communities exhibit distinct responses to varying altitude gradients (Figures 2–4). The diversity of soil bacteria significantly decreased with increasing altitude, whereas the Shannon index of fungal community exhibited no significant variation across different altitudes (Figure 2). These findings diverged from previous reports and may be attributed to the specific environmental conditions and vegetation types in the study area (Zhang et al., 2013). The Sejira Mountain region exhibited distinctive high-altitude climatic characteristics, characterized by lower temperatures and higher humidity, which may exert varying impacts on the growth of bacteria and fungi (Fu et al., 2023a,b). Moreover, the vegetation composition in this region predominantly comprised alpine meadows and coniferous forests, potentially leading to differential effects of root exudates and litter on microbial communities across different vegetation types (Fu et al., 2023a,b; Rahbek, 2005). Alpine meadow plants typically generated readily decomposable residues that facilitate rapid bacterial proliferation and enhance diversity (Condron et al., 2010), whereas coniferous forests produced recalcitrant residues abundant in lignin and cellulose, relying more heavily on fungi for decomposition processes (Li et al., 2021). Hence, these distinct environmental and vegetation characteristics may have contributed to the disparate reactions of bacterial and fungal communities to changes in altitude in this study.

**Figure 7:**
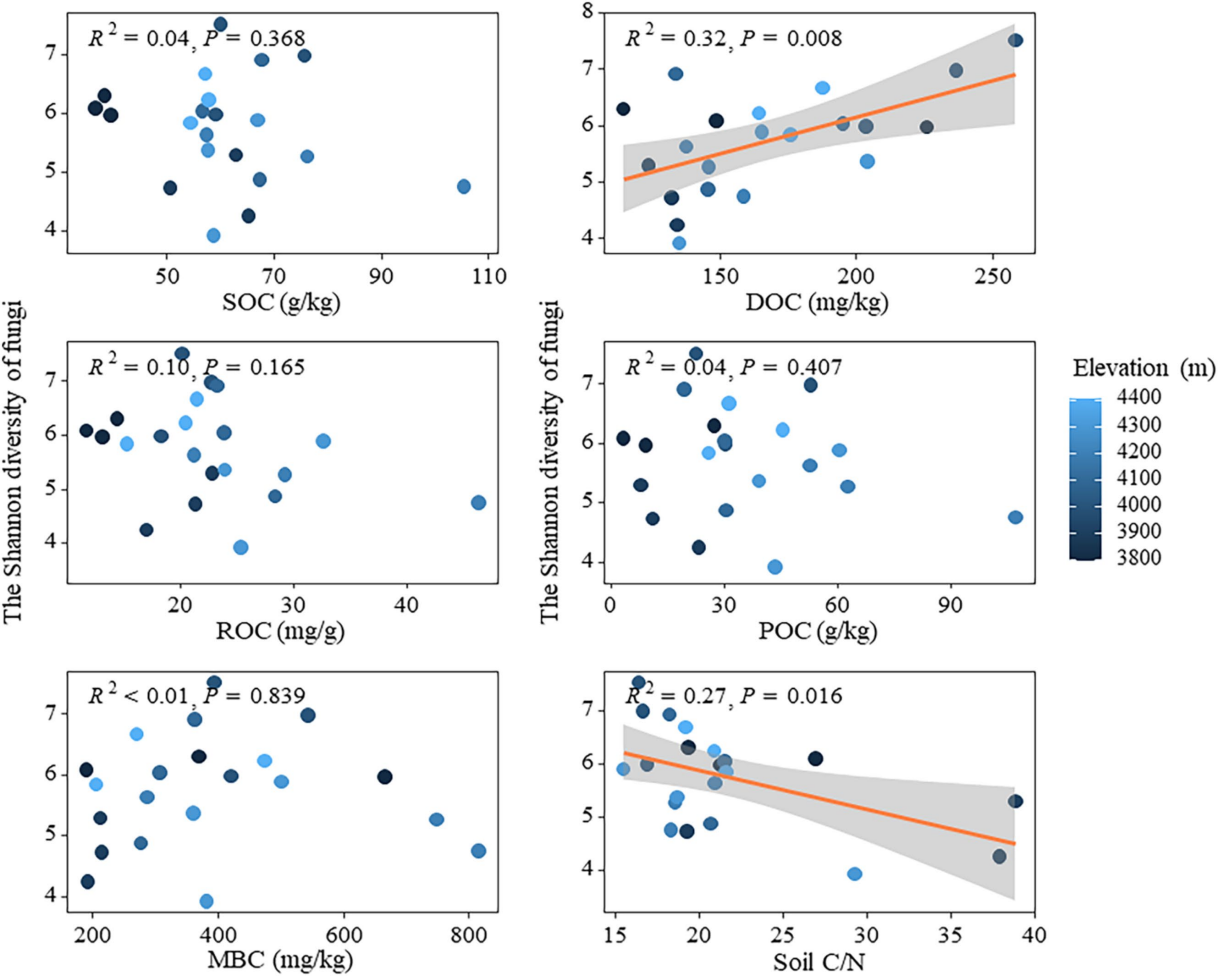
Relationships between soil active organic carbon components and fungal diversity.

In theory, microbial communities at high altitudes may face extreme survival pressures due to low temperature, aridity, and nutrient-limited soil environments (Xu et al., 2015), which typically lead to diminished microbial activity and diversity. This study demonstrated a decline in N and P content (e.g., TN, NH+ 4-N, TP, and AP) in the soil with increasing altitude (Table 1). Such reduction could impede plant growth, decrease aboveground biomass, alter carbon distribution both above and below ground, as well as diminish the input of plant litter into the soil. Consequently, this can affect the availability of easily degradable carbon sources necessary for microbial growth while indirectly influencing bacterial community diversity (Yan et al., 2018). The findings were validated through linear regression analysis, which revealed that both the ROC and POC components of soil active organic carbon had a significant negative impact on the Shannon index of bacterial community (Figure 5). The strong correlations between carbon fixation genes (cbbL and cbbM) and key soil carbon pools (SOC and ROC), along with the significant associations between degradation genes (amylase and cellulase) and POC and ROC (Supplementary Figure S4), underscore the dual role of autotrophic microorganisms in carbon sequestration and microbial decomposition in driving the accumulation of both stable and labile carbon fractions in soils. Additionally, the random forest model confirmed that altitude, as well as the ROC and POC components, played crucial roles in influencing the Shannon index of bacterial community (Supplementary Figure S5). Conversely, the presence of DOC had a substantial positive impact on fungal Shannon diversity, whereas C/N exhibited a significant negative effect on it (Figure 7). This may be attributed to the preference of fungi for complex carbon sources, such as lignin and cellulose, which influences their diversity (Hanson et al., 2008). Fungi, characterized as K-strategists, typically exhibit slower growth rates and enhanced decomposition abilities, enabling them to exploit these intricate carbon sources and maintain their ecological niche (Ciccazzo et al., 2016). Moreover, during adaptive evolution, fungi generally display slower growth rates, larger body sizes, longer life cycles, and produce fewer but more reliable offspring (Stearns, 1976), facilitating the maintenance of diversity and stability in resource-limited high-altitude environments. The random forest model revealed that despite the influence of other soil physicochemical properties, such as available phosphorus (AP), on the Shannon index of the fungal community, there was no significant decrease in fungal diversity with increasing altitude (Supplementary Figure S5). This finding suggested that fungal communities were capable of maintaining temporal diversity through specific adaptive mechanisms, even in the presence of reduced AP content. Firstly, fungi exhibited a remarkable ability to acquire nutrients at high altitudes; although AP content decreased with altitude, they can still obtain nutrients from complex substances like lignin and cellulose (Babič et al., 2017). Consequently, fungi were able to sustain their growth and diversity despite nutrient limitations. Furthermore, the symbiotic association between fungi and plant roots played a crucial role in high-altitude ecosystems. Through the formation of arbuscular mycorrhiza and ectomycorrhiza with plants, fungi acquired essential carbon sources from their hosts while facilitating nutrient uptake by plants from the soil (Read and Perez-Moreno, 2003). This mutualistic relationship not only supplemented fungal nutrient requirements but also enhanced their ability to thrive in low-nutrient environments. Fungi adapted to reduced AP content by adjusting metabolic pathways and physiological characteristics, thereby maintaining relative diversity stability as altitude increases. These findings further supported the resilience and stress resistance of fungal communities in adapting to high-altitude environments (Figure 2).

### 4.3 Impacts of altitude gradient and soil active organic carbon on microbial community composition and structure

This study investigated the soil microbial community composition and structure at different altitude gradients in the Sigera Mountain in Southeast Tibet, revealing that it is significantly affected by altitude changes and soil active organic carbon components. Acidobacteria, Proteobacteria, Chloroflexi, Verrucomicrobiota, Actinobacteriota were the dominant bacteria. As the altitude increased, there was a significant decrease in the relative abundance of Proteobacteria, while the relative abundance of Actinobacteriota exhibited an opposite trend. This reflected the adaptive differences between different bacteria to the changes in altitude (Figure 2A; Supplementary Table S2). The increase in altitude is typically accompanied by a concomitant decrease in temperature, oxygen levels, and nutrient availability, collectively influencing the composition and diversity of the microbial community (Zeng et al., 2022). Actinobacteriota, being an oligotrophic bacterium, exhibited enhanced tolerance to environmental fluctuations induced by altitude elevation (Liu et al., 2024). This could be attributed to Actinobacteriota’s efficient utilization of organic matter under conditions of limited nutrients and its ability to adapt to extreme habitats (Liu et al., 2023). Conversely, Proteobacteria, being a representative eutrophic strategy group, exhibits enhanced growth and vitality in nutrient-rich environments; hence its relative abundance diminishes in nutrient-poor high-altitude habitats (Zhang et al., 2020). Furthermore, the elevation-dependent increase in similarity among bacterial communities may be attributed to elevated levels of SOC, ROC, and POC constituents (Figures 1, 6; Supplementary Figure S3). The augmented presence of these active organic carbon components stimulated a significant rise in the relative abundance of microbial groups reliant on such carbon sources, leading to convergence in microbial community composition and structure.

The fungal community at low elevation timberlines was predominantly composed of Ascomycota (Ascomycota), while Basidiomycota (Basidiomycota) dominated at high altitudes (Figure 2B). This observation aligns with the findings reported in a previous global-scale study on temperate forests (Shu et al., 2018). The relative abundance of Mortierellomycota exhibited a significant decrease with increasing altitude. Soils characterized by low C/N ratios typically exhibited bacterial predominance, whereas soils with high C/N ratios generally displayed fungal predominance, which aligns with the findings of this study (Figures 7, 6; Supplementary Figures S5, S6). Bacteria possess the capability to rapidly utilize easily decomposable organic matter, thereby exhibiting a competitive advantage in low C/N environments (Azam et al., 1994). Conversely, fungi excel in high C/N environments owing to their proficiency in breaking down intricate and recalcitrant organic matter (Negi and Suthar, 2018). It is noteworthy that microorganisms encompass diverse heterotrophic types beyond the decomposition of organic residues, contributing to soil ecosystems through various mechanisms. As saprophytic organisms, basidiomycetes and ascomycetes are widely acknowledged as pivotal drivers of plant residue decomposition in forest ecosystems due to their exceptional biodegradability (Barrow, 1993). They efficiently convert organic matter derived from plant residues into soil, thereby providing nutrients for plants and supporting the sustained health and productivity of alpine ecosystems. However, despite functional similarities between basidiomycetes and ascomycetes, they exhibited significant differences in elevation distribution, implying that their ecological roles in nature do not completely overlap (Ren et al., 2018). Further research was warranted to investigate potential variations in their adaptations to soil nutrient conditions, as these discrepancies may give rise to fundamental disparities in their survival strategies and interactions with other organisms (Khosravi Aqdam et al., 2023; Song et al., 2015). Our findings also demonstrated that soil AP was the primary driver of fungal community dynamics across different altitudes of Sejila mountain. Moreover, AP exerted a significant influence on fungal growth and reproduction due to its essential role in cellular division and energy conversion (Bryant et al., 2008). In low-phosphorus soil environments, fungi capable of efficient phosphorus utilization may dominate, thereby altering the overall composition of the fungal community. Conversely, under phosphorus-deprived conditions, fungal communities may adapt by developing more effective mechanisms for acquiring and utilizing phosphorus (Li et al., 2022; Mganga et al., 2022). Notably, rare fungal species might exhibit heightened activity in phosphorous-rich environments, facilitating organic matter decomposition and nutrient cycling.

## 5 Conclusion

The findings of this study have significant implications for comprehending the relationship between soil carbon cycling and microbial communities in alpine ecosystems. As altitude increasing, SOC, ROC, and POC initially increased and then decreased, reaching a peak at 4200 m, which addressed our initial inquiry. This pattern may be attributed to the diminished microbial activity caused by the low temperature, low oxygen levels, and high humidity prevalent at higher altitudes. Different vegetation types and methods of carbon input influence the distribution of soil active carbon components. With increasing altitude, there was a decline in bacterial community Shannon diversity while their structure tended to become more similar. Conversely, fungal community Shannon diversity did not exhibit significant changes but displayed relatively distinct structures. This discrepancy might be due to variations in adaptability between bacteria and fungi toward environmental changes as an explanation for our second question. Bacterial community was primarily influenced by SOC, ROC, and POC, whereas soil C/N ratios along with DOC and AP were key factors affecting fungal community diversity. POC was a primary determinant influencing the composition and structure of fungal communities, potentially attributed to the enhanced adaptability of fungi enabling their survival in more challenging ecosystems, thereby addressing our third research question. Although our findings shed light on the relationship between soil active organic carbon components and microbial communities across different altitudinal gradients on Sejila mountains, further research is needed to fully comprehend how changes in altitude gradients impact the association between microbial communities and soil organic carbon dynamics.

## Data Availability

The datasets presented in this study can be found in online repositories. The names of the repository/repositories and accession number(s) can be found in the article/Supplementary material.
