# Supplementary material for: Distribution characteristics of soil active organic carbon at different elevations and its effects on microbial communities in southeast Tibet
Source: Front Microbiol. 2024 Oct 23;15:1458750. doi: 10.3389/fmicb.2024.1458750 (PMC11537981; doi:10.3389/fmicb.2024.1458750)
Supplement: Supplementary file 1 [file Data_Sheet_1.PDF]

Supplementary Material

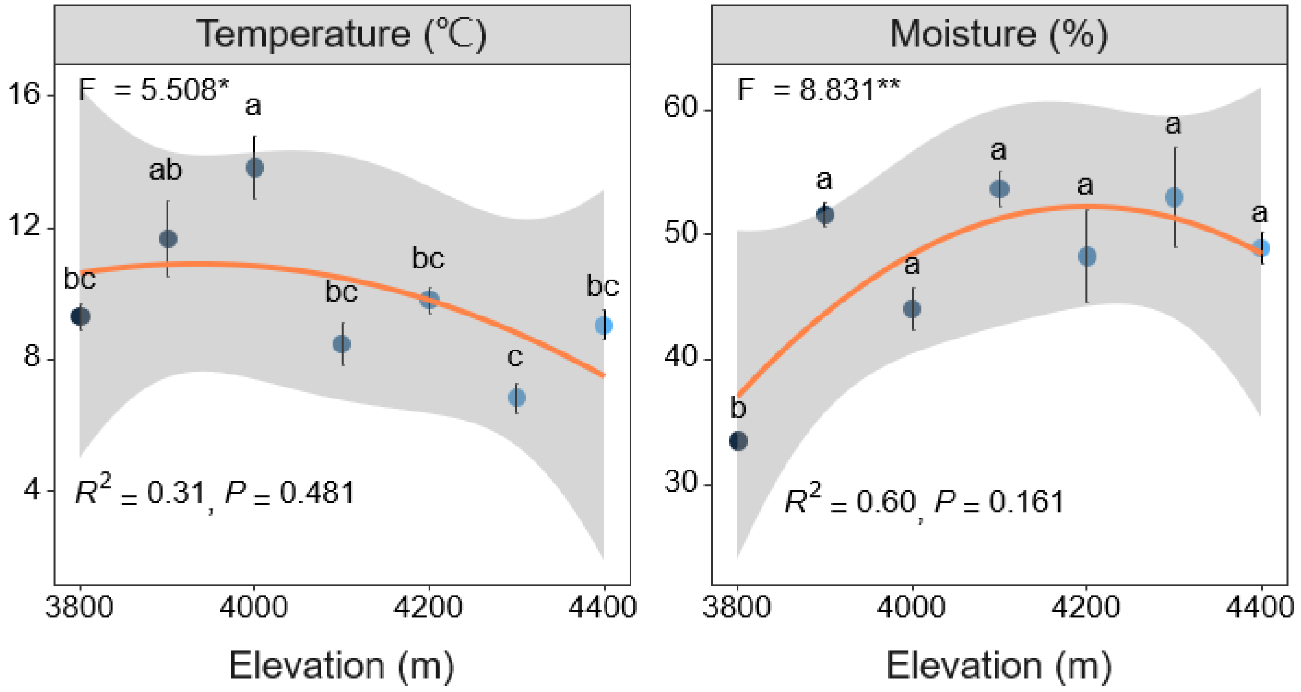

Supplementary Figure 1 Soil moisture and temperature along with elevation. The letters were obtained through one-way ANOVA (F value) followed by a post hoc test using Duncan's new multiple range test. The R^2 and P values are obtained from the fitted curve.

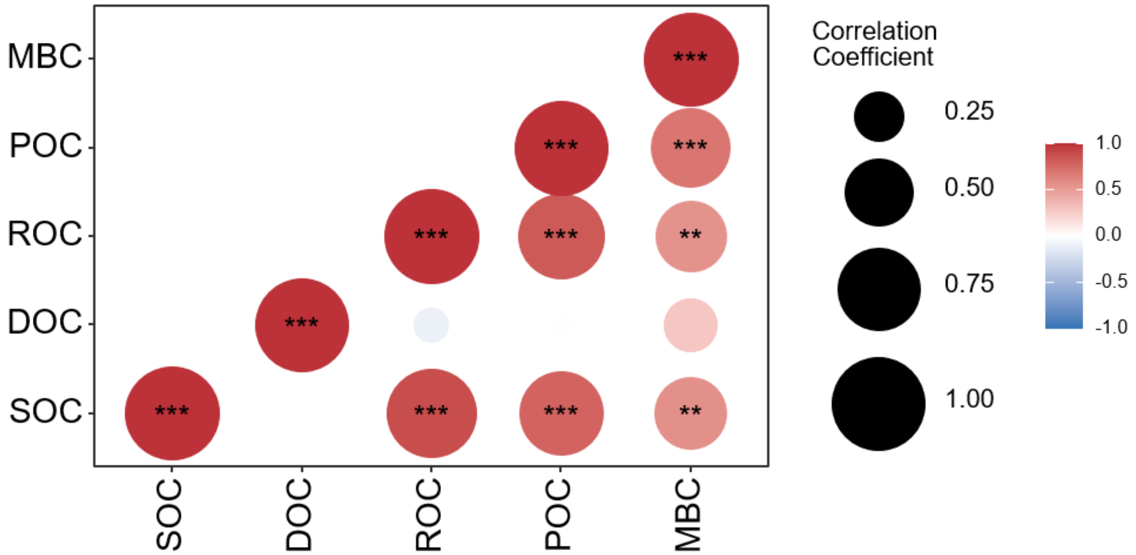

Supplementary Figure 2. Relationships among soil active organic carbon components

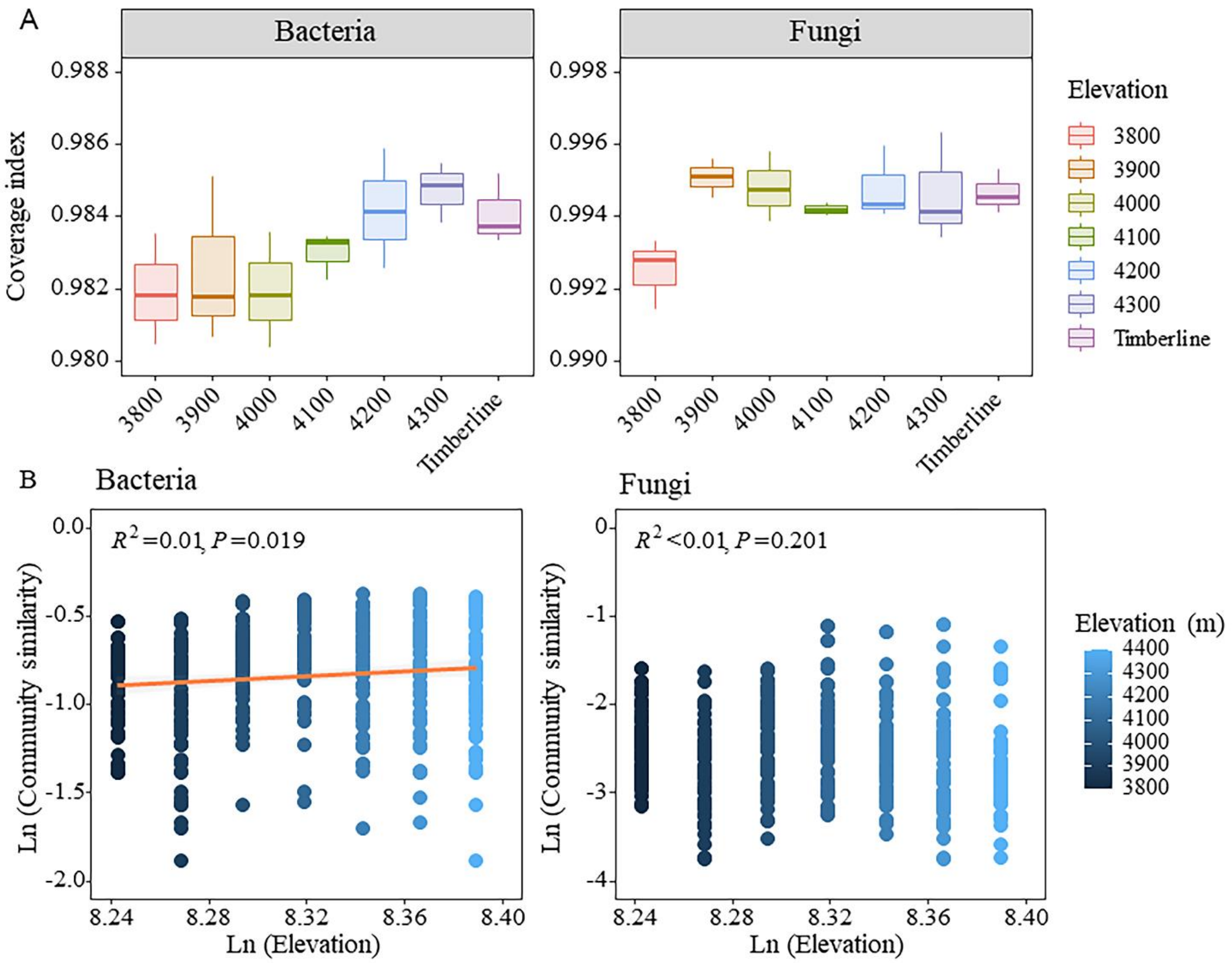

Supplementary Figure 3. Variations in soil microbial community along altitudinal gradients. (A) Coverage index and (B) Microbial community similarity

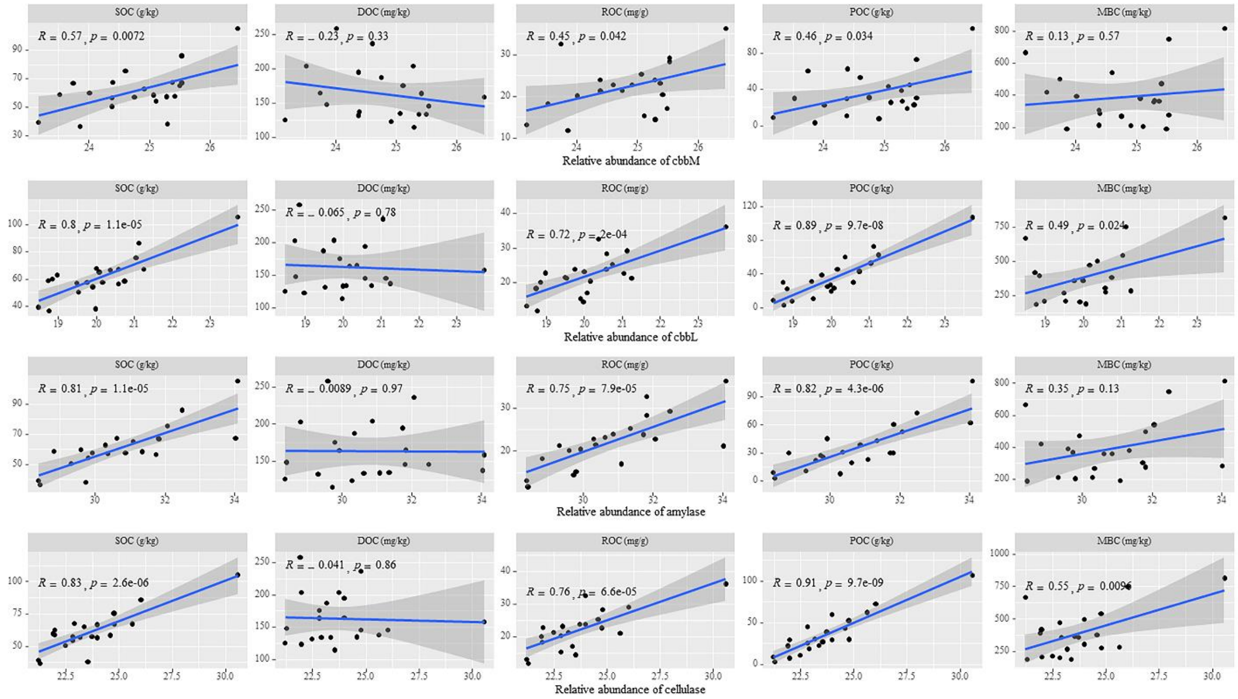

Supplementary Figure 4. Correlation between relative abundances of functional genes and soil organic carbon and its active components.

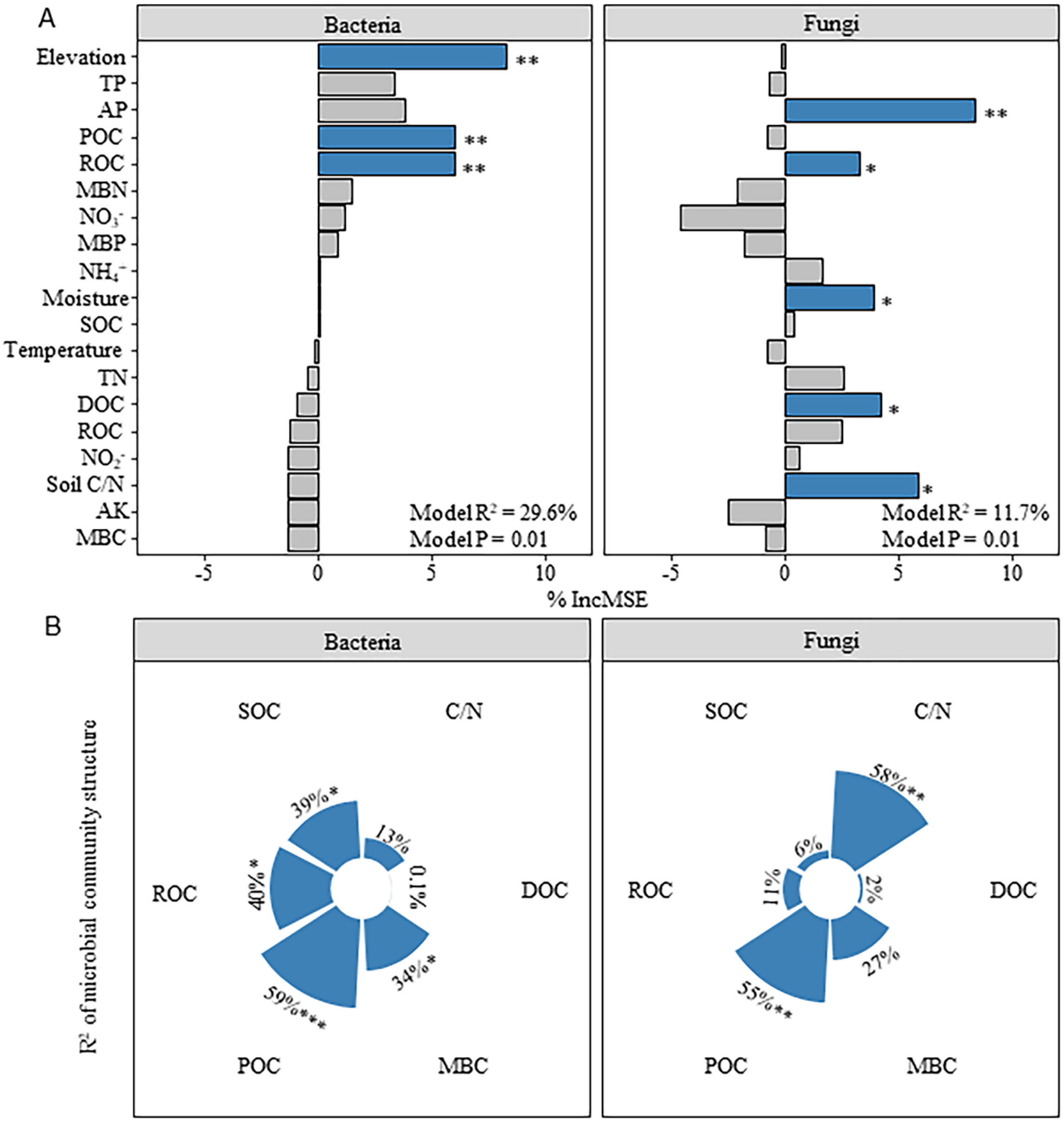

Supplementary Figure 5. Ranking the importance of environmental contributions to bacterial and fungal community diversity and composition.

(A) The results depicted in the figure were obtained through analysis using a random forest model. The gray color indicates insignificance, while the blue color signifies significance. An asterisk denotes statistical significance at the α =0.05 level, and * represents P<0.05; ** indicates P<0.01.

(B) The results depicted in the figure were obtained through analysis using CCA. An asterisk denotes statistical significance at the α =0.05 level, and * represents P<0.05; ** indicates P<0.01, *** indicates $P < 0.001$.

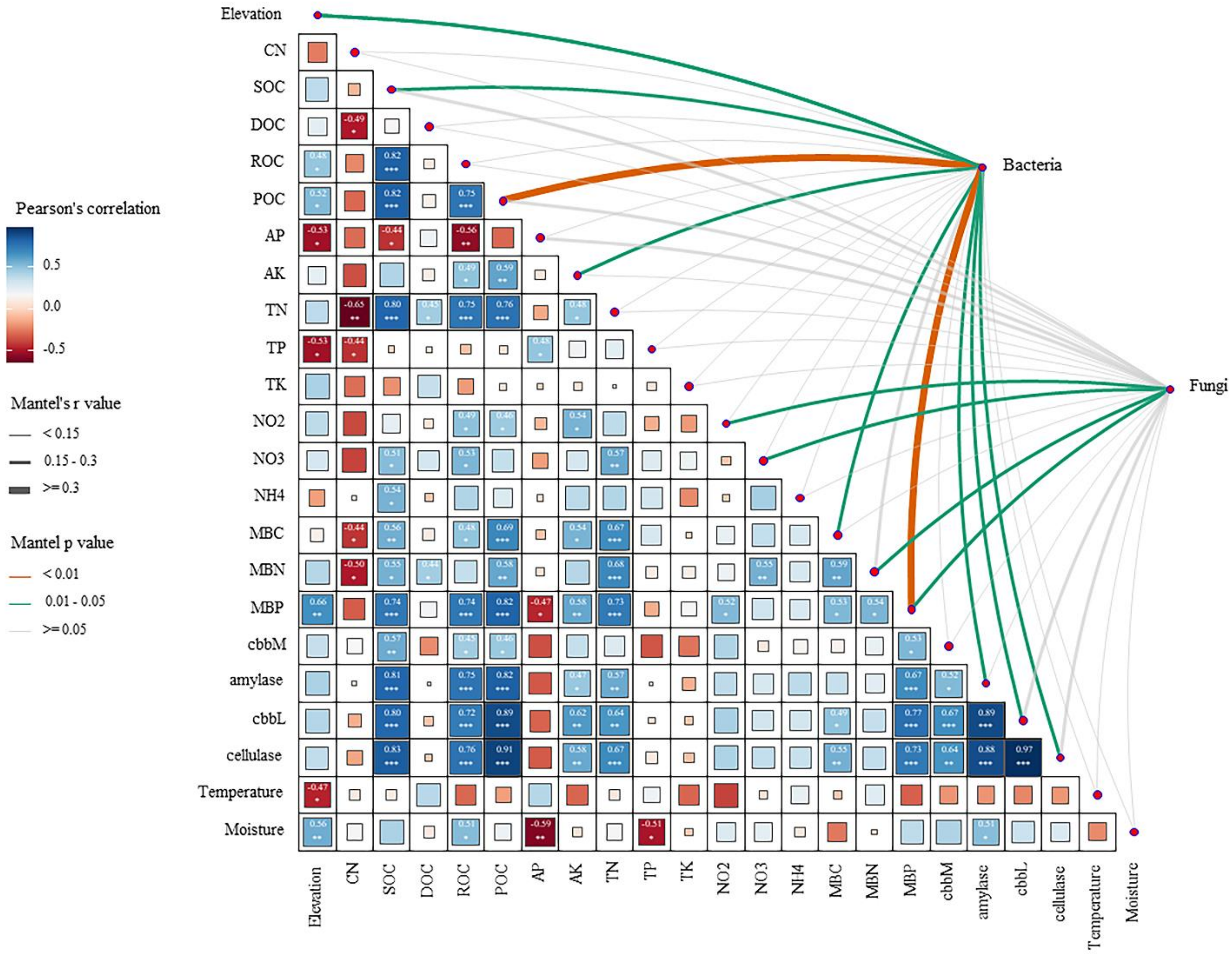

Supplementary Figure 6. Relationships between bacteria and fungi and environmental factor.

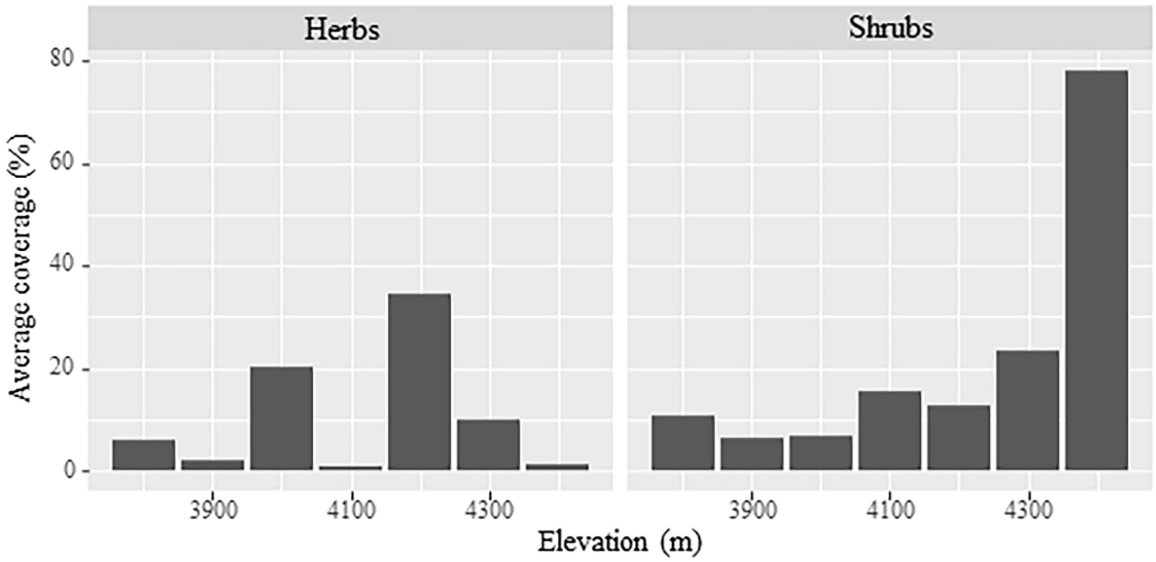

Supplementary Figure 7. Average coverage of plant along with elevation

Supplementary Table 1. Variations in soil microbial diversity along altitudinal gradients

Types	Indictors	Altitude (m)							F	P
		3800	3900	4000	4100	4200	4300	Timberline		
Bacteria	Shannon	10.43±0.17a	10.11±0.18abc	10.37±0.01a	10.32±0.14ab	10.01±0.17bc	9.83±0.29c	9.97±0.18c	4.81	0.01*
	Chao1	6591±377a	6008±616abc	6574±334a	6397±284.33ab	5861±470abc	5574±422c	5780±244bc	2.97	0.04*
	Richness	5648±306a	4975±513a	5636±315a	5537±287a	5000.33±426.86a	4670±437b	4878±212ab	3.57	0.02*
Fungi	Shannon	6.12±0.17ab	5.04±0.99b	6.83±0.78a	5.95±1.03ab	5.22±0.44b	5.06±1.02b	6.25±0.42ab	2.45	0.08
	Chao1	1902±132a	1440±123b	1622±71ab	1522±111b	1334±277b	1312±332b	1512±90b	3.42	0.03*
	Richness	1422±74a	1069±139ab	1276±118ab	1083±156ab	985±185ab	944±249b	1141±82ab	3.53	0.02*

Note: Different lowercase letters indicate that there are significant differences between indicators at different sampling altitudes ($P<0.05$), $^*P<0.05$. Data are mean±standard deviation (n=3).

Supplementary Table 2. Alterations in the relative abundance of different taxonomic groups within bacterial and fungal communities

Types	Indictors	Altitude (m)							F	P
		3800	3900	4000	4100	4200	4300	林线		
Bacteria	Acidobacteriota	43.71%	43.41%	48.27%	46.94%	55.64%	52.55%	48.98%	0.32	0.58
	**Proteobacteria**	**32.52%**	**30.53%**	**27.56%**	**26.35%**	**24.57%**	**24.45%**	**22.42%**	**25.27**	**0.001*** **
	Chloroflexi	7.44%	8.19%	7.12%	8.26%	6.18%	7.52%	10.27%	0.01	0.92
	Verrucomicrobiota	4.58%	5.26%	4.77%	4.60%	2.89%	3.47%	4.76%	1.79	0.20
	**Actinobacteriota**	**1.89%**	**2.00%**	**2.00%**	**2.65%**	**2.34%**	**3.35%**	**2.86%**	**5.72**	**0.03***
	Gemmatimonadota	1.46%	2.23%	2.13%	2.89%	1.67%	1.85%	2.12%	0.02	0.90
	Bacteroidota	2.27%	1.41%	1.66%	2.32%	1.51%	1.66%	2.07%	0.36	0.56
	RCP2-54	1.00%	1.02%	1.43%	1.17%	1.32%	1.24%	1.23%	0.08	0.79
	**WPS-2**	**0.33%**	**0.41%**	**0.74%**	**1.20%**	**0.77%**	**1.47%**	**1.18%**	**10.22**	**0.01****
	Myxococcota	0.58%	0.54%	0.66%	0.72%	0.72%	0.59%	1.02%	2.62	0.12
Fungi	Basidiomycota	53.71%	72.14%	49.60%	69.46%	64.80%	46.27%	28.59%	4.18	0.06
	Ascomycota	29.15%	15.02%	23.05%	12.84%	23.63%	48.69%	60.72%	3.74	0.07
	**Mortierellomycota**	**11.70%**	**2.75%**	**5.95%**	**2.73%**	**4.40%**	**1.86%**	**2.96%**	**9.18**	**0.01****
	Rozellomycota	0.38%	0.71%	4.57%	1.71%	1.62%	0.07%	0.06%	0.78	0.39
	Mucoromycota	0.05%	0.10%	0.07%	0.09%	0.01%	0.01%	0.02%	3.47	0.08

Notes: The bold representation of ANOVA has significant differences.

Supplementary Table 3. Results of PERMANOVA based on Bray-Curtis distances testing the effect of altitude on microbial community structure

Microbes	Factor	Df	Sum Sq	R^2	F	P
Bacteria	Elevation	6	1.370	0.424	1.719	0.007**
	Residual	14	1.859	0.576		
	Total	20	3.229	1.000		
Fungi	Elevation	6	3.457	0.417	1.666	0.001***
	Residual	14	4.840	0.583		
	Total	20	8.297	1.000		

Notes: The table presents the results of the PERMANOVA (Permutational Multivariate Analysis of Variance) analysis based on Bray-Curtis dissimilarity, testing the influence of altitude on bacterial and fungal community structures. The analysis was conducted on microbial OTU data from soils sampled along an altitudinal gradient. The table shows the degrees of freedom (Df), sum of squares (Sum Sq), explained variation (R^2), F-statistics (F), and p-values (P) from the PERMANOVA test. Significant p-values ($P < 0.05$) indicate a statistically significant effect of altitude on the microbial community structure. The R^2 value represents the proportion of the variance in microbial community structure explained by altitude.
